# An Iterative Error Correction Procedure for Single Sheet Testers Using FEM 3D Model

**DOI:** 10.3390/s25123813

**Published:** 2025-06-18

**Authors:** Robert Krobot, Martin Dadić

**Affiliations:** 1 DEIF A/S, Frisenborgvej 33, 7800 Skive, Denmark; 2 Faculty of Electrical Engineering and Computing, University of Zagreb, Unska 3, 10000 Zagreb, Croatia; martin.dadic@fer.unizg.hr

**Keywords:** core losses, BH curve, single sheet tester, FEM 3D analysis, iterative procedures, electrical steel, steepest descent

## Abstract

Determination of single-valued BH curve and power loss curve of electric steels is an important parameter in the design of electrical machines and transformers. This paper proposes a correction procedure for the measurement of anhysteretic BH curve and power losses, based on the finite element model (FEM) and SST apparatus. A 3D finite element model (FEM) of the SST (Single Sheet Tester) was developed with respect to the IEC 60404-3 standard. The measurement results obtained with a standardized SST apparatus are fed to its FEM and used to iteratively correct initial BH and power loss curves, obtained using magnetic equivalent circuits theory. The proposed iterative correction procedure is based on the steepest descent algorithm, while the stopping criteria were based on the difference between simulated and measured global variables (power loss, induced voltage, and primary current). After correction, root mean squared errors were decreased from 1.85 A/m to 42.88 × 10^−3^ A/m for the BH curve, and from 44.5 × 10^−4^ W/kg to 7.28 × 10^−4^ W/kg for the power loss curve.

## 1. Introduction

Determination of BH- and power loss curves is an important parameter in the design of electrical machines and transformers [1]. While incorporating hysteresis in numerical models gives an advantage in the accurate modeling and calculation of such devices, it increases calculation time, and single-valued models of the BH curve are still needed both in analytical calculations using magnetic equivalent circuits modeling [2,3,4,5] and numerical calculations. The single-valued normal BH curve is typically measured using the locus points of the symmetric hysteresis cycles [6]. Two standardized methods have been developed for the magnetic curve and core loss measurement in the electrical steel at power frequencies up to 400 Hz: the Epstein frame (EF) and the Single Sheet Tester (SST). The measurement method used in EF is defined by the IEC standard 60404-2; 1996 [7], which is widely used as the main measurement method for the electrical steel sheets. The analysis with the Epstein frame is performed by applying the classic analytical theory of the transformer, and is an approximate calculation due to the numerous approximations involved. Anisotropy and the corner effects are both neglected, as well as the non-homogeneous distribution of the magnetic field. It is known that the measurement system is not able to evaluate the true local hysteresis cycle and, consequently, the true specific power loss [8]. The SST measurement method is defined in IEC standard 60404-3 [9]. The concept of SST assumes the vanishing influence of the magnetizing yoke system [10,11]. While both EF and SST measurement methods are standardized, they apply a magnetic equivalent circuits approach and analytical theory, thus introducing errors in the measurement of anhysteretic BH curve and power losses, since the assumption of uniform distribution of magnetic field and magnetic flux density across the sheet sample(s) is are approximation. Both in the Epstein apparatus and the SST apparatus, the anhysteretic BH curve and power loss curve are estimated using global variables, e.g., induced voltage in the secondary winding and electrical current in the primary winding. In this way, applying Ampère’s law, the magnetic field is linearly related to the primary current (measured using a current shunt), and the magnetic flux density is calculated from the time derivative of the induced secondary voltage. Thus, if the mentioned measurement systems are modeled and simulated in FEM environment and fed by the BH curve and power loss curve obtained using the same experimental setup, the results are not consistent with measured data, especially at high magnetic flux densities close to the saturation region.

In [12], an analysis of simulation results of the Epstein apparatus and SST apparatus is presented using simulated global variables, in comparison with the “true” BH curve and locally calculated losses. Generally, “true” BH curves possess bigger magnetic flux densities B for the same values of magnetic fields H, and locally calculated power losses are higher as well. In [13,14] is presented a comparison of the Epstein frame and SST is presented. It can be observed that the values of B and H in the tested sample are not constant along the path of the magnetic flux. The SST measurement system provides a better uniformity of B and H than the Epstein Frame, because the sample under test has almost uniform excitation. The SST is more accurate, but the magnetic losses errors cannot be neglected. The loss models are normally integrated with the finite element method, which is generally used to analyze the magnetic field distribution of electric machines [15,16].

Numerous research was conducted in FEM environment where various types of iterative procedures were proposed to obtain representative BH curves and core losses. A method for core loss calculation that considers anisotropy is proposed in [17]. The specimen under test is usually sheared into strips along the rolling direction, and the measurements are carried out in the same direction. Here, the convergence criterion $\left\|\Theta_{B}^{k+1}{-\Theta }_{B}^{k}\right\|\leq 2{}^{\circ}$ was considered for each iteration k, where $\Theta_{B}$ stands for the angle of B, and 0° denotes the angle of the rolling direction.

In [18], a numerical method for the calculation of core losses in synchronous and DC machines is presented. The calculation is based on FEM analysis, which provides flux density waveforms and Fourier analysis.

The calculation of core losses in SiFe laminations under DC bias and harmonics based on the Jiles–Atherton (J-A) dynamic hysteresis model, combined with finite element analysis, is presented in [19]. The dynamic model combines the traditional J-A hysteresis model with the models of instantaneous eddy current and excess losses, where the dynamic parameters were identified considering the influences of DC bias on excess loss. The proposed model was experimentally verified by comparing total core losses and dynamic hysteresis loops under magnetizations with DC biases equal to 40 A/m, 80 A/m, and 120 A/m with different flux densities. The study shows that the implemented dynamic J-A model in FEM environment accurately calculates the core losses in SiFe laminations under various excitations and the optimum design and accurate performance prediction can be achieved.

The 3D finite element computations and measurements were used in [20], where the effect of interlaminar contacts on the equivalent conductivities of the EI core was studied. The deduced specific eddy current loss from the measurement is used to obtain the equivalent conductivities using an iterative approach. These equivalent conductivities were used to model the laminated sheets by using the A-V formulation that involves both the magnetic vector potential A and the electric scalar potential V and aiming to balance the accuracy while minimizing computation time. The core loss of the EI core is measured, and the eddy current loss is extracted from the measurements. Based on the eddy current loss, the equivalent conductivities are determined using an iterative approach.

In [21] an iterative procedure for the measurement of small-signal local permeability of electrical steels, based on measurements and FEM is presented. The initial permeability is iteratively adjusted based on halving the initial increment until error tolerance is achieved. In [22,23,24], the iterative procedures for reconstruction of BH curves based on measurements at transformers, rotating machines, or nonstandard EI cores are described. The SST apparatus, as well as the Epstein frame (EF), is used for the quality control of electrical steel. Both methods are standardized, thus ensuring the repeatability of the measurement results and unified methods for the preparation of the samples. On the other side, there is a known difference between the actual properties of electric steels and measurement results of SST and EF [12,13,14]. In a more accurate calculation, needed in research, optimization, and design of electromagnetic devices, a correction method for the measurement results would be beneficial, thus increasing the quality and reliability of the products. It is also possible to a posteriori measure the properties of electrical steel, using manufactured machines or devices. In such cases, the measurements also differ from the “true” properties of the materials. Known methods for the correction of the measurements performed on manufactured devices are [21,22,23]. In this paper, we propose a correction method using measurements on steel samples, performed with the SST apparatus prior to the design and manufacturing. The corrected results can be further applied to a more accurate design and optimization. Thus, the main idea of this paper is to introduce a correction procedure for the anhysteretic BH curve and power losses, measured using an SST apparatus. The measurement results, obtained with a standardized SST apparatus are fed to a FEM (finite element method) model of the SST apparatus, and used to iteratively correct initial curves to fit the measurements. While single-valued and power loss functions are naturally monotonic curves, they are measured in discrete points, and the proposed correction procedure is also obtained in these discrete points.

The paper is organized as follows: In Section 2, the core loss models are discussed. Section 3 gives an overview of BH curve models applied in FEM. The proposed simulation setup of the FEM 3D model is given in Section 4, while the iterative procedure for the correction of the errors related to the BH and PB curves is given in Section 5. The results are presented in Section 6, and the conclusions are in Section 7.

## 2. Core Loss Models

The core loss data for the same electrical steel varies between the manufacturers. The magnetic properties of the materials are affected by mechanical processes like cutting, punching, welding, and stacking, especially in the range 0.4 T to 1.5 T [25,26,27,28]. The technical data of the electrical steel and the accurate measurements are important for precise results. Hence, various core loss models were developed for core loss estimation. In general, these models could be divided into three main categories:

1. Steinmetz equation-based models.
2. Loss separation-based models.
3. Mathematical hysteresis-based models.

### 2.1. Steinmetz Equation-Based Models

The first method for iron loss calculations was proposed by Charles P. Steinmetz in 1892 [29]. The core losses were defined as an exponential function depending on the peak magnetic flux density and the frequency as follows in (1):

(1) $$P_{fe}={C_{SE}f}^{\alpha }{\hat{B}}^{\beta }$$

where $P_{Fe}$ is the time-averaged iron loss per volume, f is the frequency, $\hat{B}$ is the peak magnetic flux density and $C_{SE}$, α and β are the Steinmetz coefficients are determined by fitting the model to the loss measurement data and are highly frequency dependent. This method is only valid for sinusoidal excitation and for a certain frequency range and flux density levels.

The high-frequency harmonics often exist in modern devices and machines, and the magnetic flux is commonly distorted and non-sinusoidal. In such an environment, the mentioned model could not be used. To overcome this challenge, models are extended, and one of these is the Modified Steinmetz Equation (MSE) [30]. The equivalent frequency that is here introduced in (2) is a function of the macroscopic re-magnetization rate [31], which is proportional to the rate of change of the flux density dB/dt.

(2) $$f_{eq}=\frac{2}{∆B^{2}\pi^{2}}\int_{0}^{T}{\left(\frac{dB}{dt}\right)}^{2}dt$$

where ∆B is the difference between the maximum and minimum flux densities in one cycle of magnetization. From (1) and (2), a new equation could be extracted, and core losses could be calculated as follows in (3):

(3) $$P_{fe}=C_{SE}f_{eq}^{\alpha -1}{\hat{B}}^{\beta }f_{r}$$

where $f_{r}$ is the re-magnetizing frequency, $C_{SE}$, α and β, are the fitting coefficients. In this method, the accuracy decreases with very small variations in the fundamental frequency. The improved model of MSE is the Generalized Steinmetz Equation (GSE) [32]. Here, the core losses are calculated using the flux density and the rate of change of flux density as:

(4) $$P_{fe}=\frac{1}{T}\int_{0}^{T}C_{SE}{\left|\frac{dB}{dt}\right|}^{\alpha }{\left|B(t)\right|}^{\beta -\alpha }dt$$

where T is the period. The advantage of the GSE is that the DC pre-magnetization is considered in the model without requiring a correction factor or measurements. The equivalent frequency and equivalent amplitude can be derived with the GSE and can be applied to the classical Steinmetz model. The accuracy of the GSE is reduced in cases when more than one peak appears throughout the period, and especially when the third, or harmonic close to the third harmonic, becomes significant [33]. The mentioned problem is resolved with improved Generalized Steinmetz Equation (iGSE) [34]. Here, a recursive algorithm separates the flux waveform into minor and major loops, and the iron loss is calculated separately as follows in (5).

(5) $$P_{fe,x}=\frac{1}{T}\int_{0}^{T}C_{SE}{\left|\frac{dB}{dt}\right|}^{\alpha }{\left|∆B\right|}^{\beta -\alpha }dt$$

where ∆B is determined for each minor loop.

In [35], a method called Natural Steinmetz Equation is introduced and is mostly focuses on triangular waveforms [36]. The waveforms are not split into major and minor loops, but the equation applies to the full period:

(6) $$P_{fe}={\left(\frac{∆B}{2}\right)}^{\beta -\alpha }\frac{C_{SE}}{T}\int_{0}^{T}{\left|\frac{dB}{dt}\right|}^{\alpha }dt$$

The main disadvantage of the Steinmetz models is the variation in the Steinmetz parameters with frequency and DC pre-magnetization.

### 2.2. Loss Separation Models

The Loss Separation Model [32] is an extended version of the Steinmetz model where the core loss is separated into hysteresis losses and dynamic eddy current losses, and it is presented in (7):

(7) $$P_{fe}=P_{h}+P_{c}=K_{h}{\hat{B}}^{2}f+K_{c}{\left(\hat{B}f\right)}^{2}$$

where $P_{h}$ is the average hysteresis loss per volume, $P_{c}$ is the average dynamic eddy current loss per volume,$\;K_{h}$ and $K_{c}$ are the loss coefficients. At low frequencies, the hysteresis loss is proportional to the hysteresis loop of the magnetic material, while the dynamic eddy current loss is calculated as follows in (8):

(8) $$P_{e}=\frac{d^{2}{\left(\frac{dB(t)}{dt}\right)}^{2}}{12\rho \gamma }$$

where d is the thickness of the lamination, ρ is resistivity, and γ is the density of the material. Further, the model can be extended with excess loss as it is presented in [37] and shown in (9):

(9) $$P_{fe}=P_{h}+P_{c}+P_{e}=K_{h}{\hat{B}}^{2}f+K_{c}{\left(\hat{B}f\right)}^{2}+K_{e}{\left(\hat{B}f\right)}^{1.5}$$

where $P_{e}$ is the average excess loss per volume, and $K_{e}$ is the excess loss coefficient.

A statistical model was developed by Bertotti [24]. The loss factor $K_{e}$ was determined by the physical description of the active magnetic objects and the domain wall motion [38,39] as follows in (10):

(10) $$K_{e}=\sqrt{SV_{0}\sigma G}$$

where S is the cross-sectional area of the steel sheet, $V_{0}$ is the statistical distribution coefficient that characterizes the local coercive field distribution based on the grain size. The conductivity is denoted with σ and G is the eddy current damping factor. The model is valid only if the skin effect is neglected [40]. The analysis of the coefficients in the mentioned statistical model is presented in [41].

### 2.3. Core Loss Calculation in ANSYS Maxwell

The finite element method (FEM) is implemented in ANSYS Maxwell 17.2.0 software which was used for the modeling of the SST. The calculation method that is used to calculate core losses in electrical steel is based on the loss separation model proposed by Bertotti in [42].

Total core loss is calculated according to (9) in a more general form, with a change in coefficient α:

(11) $$P_{FE}=P_{h}+P_{c}+P_{e}=K_{h}{\hat{B}}^{\alpha }f+K_{c}{\left(\hat{B}f\right)}^{2}+K_{e}{\left(\hat{B}f\right)}^{1.5}$$

The coefficient α is related to the specific structural properties of a ferromagnetic material. According to the [43] calculation is performed as follows: coefficient α is defined as α  = 2, while coefficient $K_{c}$ can be calculated as shown in (12):

(12) $$K_{c}=\pi^{2}\sigma \frac{d^{2}}{6}$$

where σ is electrical conductivity, d is steel sheet thickness. The following equations are implemented as follows in (13) and (14):

(13) $$K_{1}=K_{h}f+K_{c}f^{2}$$

(14) $$K_{2}=K_{e}f^{1.5}$$

To obtain the values for $K_{1}$ and $K_{2}$, the program minimizes the following polynomial (15):

(15) $$error\left(K_{1},K_{2}\right)=\sum_{i}{\left[P_{i}-(K_{1}B_{i}^{2}+K_{2}B_{i}^{1.5})\right]}^{2}=min$$

where $P_{i}$ and $B_{i}$ are the loss and magnetic induction values at the i-th ($B_{i}^{⁠},\;P_{i})\;$ pair, taken from the loss curve, that must be specified. Afterwards, the hysteresis coefficient and the coefficient of the additional losses are calculated as follows in (16) and (17):

(16) $$K_{h}=\frac{K_{1}-K_{c}f_{0}^{2}}{f_{0}}$$

(17) $$K_{e}=\frac{K_{2}}{f_{0}^{1.5}}$$

where $f_{0}$ is frequency for the specific loss curve. Some recent analyses and applications of the Bertotti model are presented in [44,45,46,47,48,49,50].

To simulate the characteristics of electrical steel in a model, the actual physically measured values must be inserted into the program. To fulfill all the conditions for the simulation, it is necessary to give a mass density, steel sheet thickness, electrical conductivity, frequency, BH, and PB curves. According to the values mentioned, core loss coefficients are calculated according to the specified values. During the simulation, the program does not use the measured values of the BH curve but instead uses interpolated curves.

The non–linear magnetic characteristics of the electrical steel sheet cause distortion of the magnetic field under high magnetic induction, resulting in larger calculation errors [51].

## 3. BH Curve Models

Analytical expressions used for the single-valued representation of a BH curves are based on simple formulas [52,53,54], or power series representation [55,56], include representation by hyperbolas [55], transcendental functions [55], exponential series or sums of exponentials [57,58], rational functions [59,60,61] and Fourier series [55,62]. The Jiles-Atherton model of hysteresis typically applies the Langevine function for the anhysteretic part of the model. Anhysteretic functions for the Jiles-Atherton model are discussed in [63], which presents a set of simple functions that can replace the Langevin function, usually applied as the anhysteretic part in the Jiles–Atherton model. In [64], successful ways to the inverse Langevin function are presented. In [65], based on the Froelich equation, a series form with additional degrees of freedom is proposed, with some refinements in [66]. As well, in [65,66], a measurement setup for toroidal cores with a more uniform magnetic flux distribution is proposed. In [67], anhysteretic curves are modeled using Bézier curves, while in [68] the nonlinear BH curve is represented by a complex exponential series. In [69], a monotonicity-preserving interproximation using spline functions and a smoothing functional is proposed.

When applied in numerical finite-element analysis (FEA), function models *f*(*s*) of anhysteretic BH curves have to satisfy the following criteria [69]:

- The model has to be continuously differentiable
- $f\left(0\right)=0$
- $f\prime \left(s\right)\geq \mu_{0}$ for every *s*
- $\underset{s\to \infty }{\lim}f\left(s\right)=\mu_{0}$

where $\mu_{0}$ denotes the magnetic permeability of vacuum. Some of the widely applied models, which satisfy these criteria, are based on cubic Hermite polynomials [70,71], while monotonicity-preserving C^l^-splines were also applied [72,73].

Mathematical hysteresis models [74] are based on micro-magnetics theory and methods of curve fitting. These models consider the real physical phenomena of the material and are complex in computation. Their accuracy depends on the provided data points of hysteresis loops [75]. Most applied models are those of Preisach and Jilles-Atherton [76,77,78,79,80,81,82,83,84,85,86,87].

**Figure 1:**
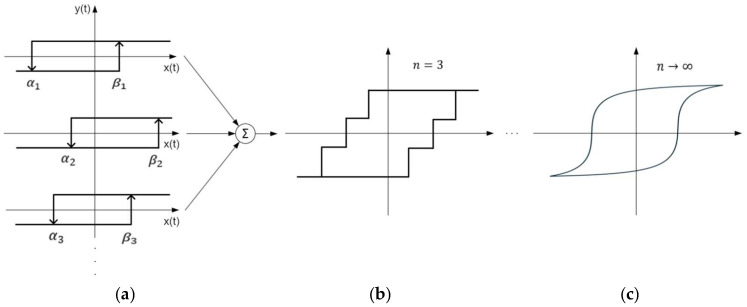
Preisach hysteresis model: The figure (**a**) shows the hysteresis modeled with 3 hysterons, while on (**b**) relay hysterons are summed up. Figure (**c**) shows the hysteresis when *n* → ∞.

The Preisach model describes hysteresis as a parallel connection of independent relay hysterons and reproduces the hysteresis curve from an infinite number of parameters, as it is shown in the following Figure 1.

## 4. Simulation Setup of the 3D SST Model

### 4.1. Specifications of the SST 3D Model

In the sequel, we explain the configuration and provide the specifications of the corresponding physical model of the SST. Respecting the mentioned IEC standard, a FEM 3D model of the SST was developed and was integrated as a transient co—simulation in Simplorer circuits. IEC 60404-3:2022 standard [9] defines the following key specifications:

(a) Yoke specifications:
  - Pole face width: 25 mm ± 1 mm
  - Coplanarity of pole faces: Within 0.5 mm
  - Gap between opposite pole faces: Not exceeding 0.005 mm
  - Yoke height: 90 mm to 150 mm
  - Yoke width: 500 mm ± 55 mm
  - Inside length of yoke: 450 mm ± 1 mm
(b) Windings specifications
  - Minimum length: 440 mm
  - Former dimensions
  - Length: 445 mm ± 2 mm
  - Internal width: 510 mm ± 1 mm
  - Internal height: 5 mm (−0/+2 mm)
  - Height: ≤15 mm
(c) Primary winding options:
  - Multiple coils: Identical coils with identical turns and connected in parallel
  - Single continuous winding: Spanning the full length
  Note: Secondary winding turn number is based on the measurement device.
(d) Test specimen specifications:
  - Length: ≥500 mm
  - Width: As wide as possible (minimum 60% of yoke width)
  - Cutting tolerances:
  - Grain-oriented steel: ±1°
  - Non-oriented steel: ±5°
  Note: Specimens must be free of burrs, flat, and without mechanical distortions.
(e) Power supply requirements:
  - Voltage and frequency stability: ±0.2%
  - Waveform: Should be sinusoidal with a form factor of 1.111 ± 1%
  Note: A stable power supply with low internal impedance is required.

**Figure 2:**
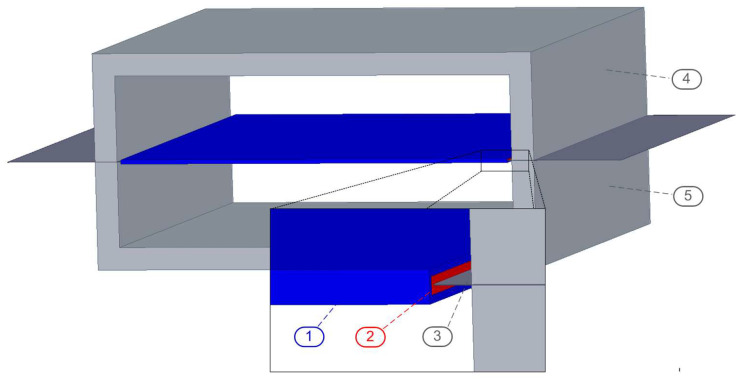
Three-dimensional model of SST; 1—primary coil, 2—secondary coil, 3—electrical steel sheets, 4—upper yoke, 5—lower yoke.

The measured magnetic induction is the sum of the flux in the sample and in the air [88,89]. To eliminate this effect, an air flux compensator was built [90]. Calibration of the air flux compensator is performed by adjusting the number of turns of the secondary coil, while the number of turns in the primary coil is fixed. The compensator is adjusted when the excitation voltage is present, the induced voltage in the secondary coil is close to zero, and the sample is not inserted in the apparatus. The FEM 3D model can be seen in Figure 2. The parameters of the simulated device can be seen in Table 1.

**Figure 3:**
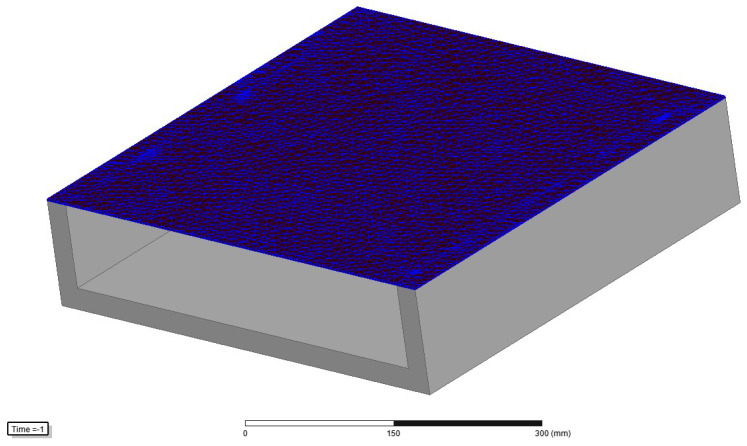
Visualization of the finite element mesh (detail)—electrical steel region.

**Figure 4:**
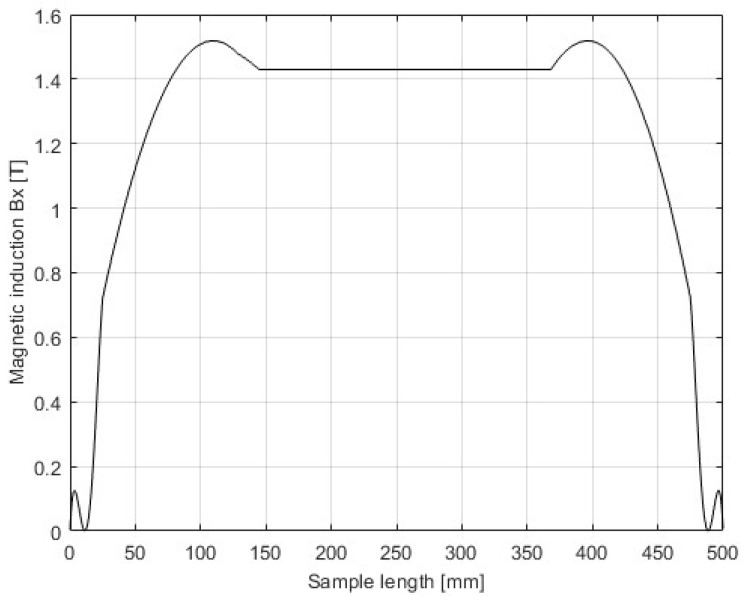
Magnetic flux density distribution—length.

**Figure 5:**
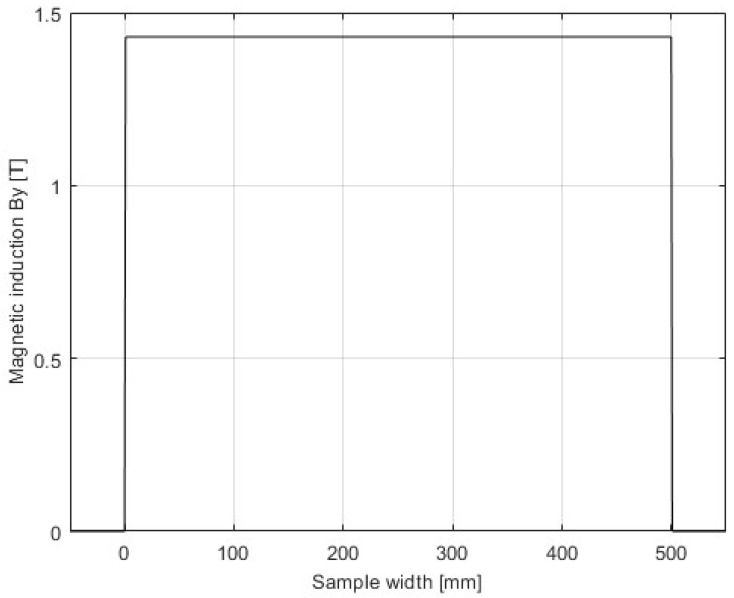
Magnetic flux density distribution—width.

**Figure 6:**
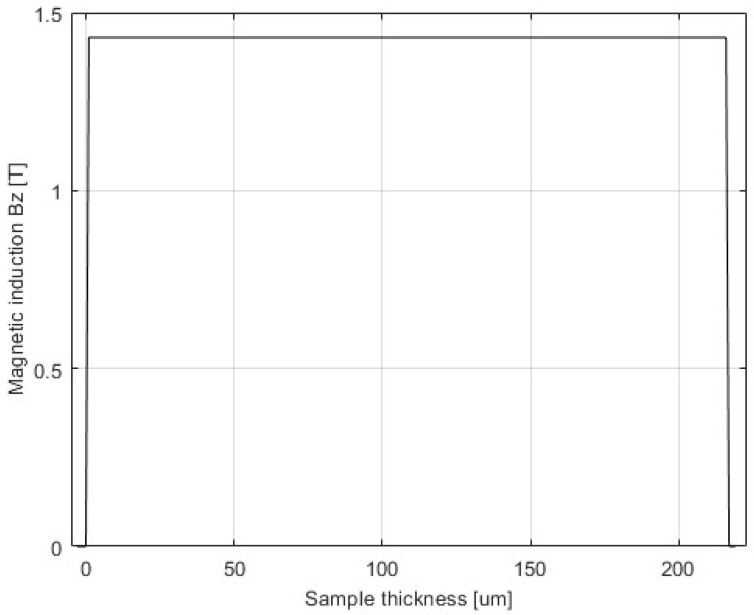
Magnetic flux density distribution—thickness.

The domain of the FEM is a rectangular box with dimensions 2500 mm × 8320 mm × 1200 mm, consisting of regions representing the device and an air box. The air box is automatically generated around the SST, using an offset of 200%. At the boundaries, the “insulating boundary” is assigned. This imposes Neumann boundary conditions with the additional constraint that the electrical current cannot cross the boundary. Therefore, the flux is tangential at the boundaries, which is satisfactory considering the distances between the boundaries and the edges of the SST. The model was validated by the inspection of the field uniformity (presented in Figure 3, Figure 4, Figure 5 and Figure 6), using a very dense mesh of finite elements. The detail of the finite element mesh (in the region of the electrical sheet sample) is visualized in Figure 3. Figure 4, Figure 5 and Figure 6 present the distribution of the magnetic flux density along the length and across the width, and the thickness of the sheet. The graphs representing magnetic flux distributions along the length and across the width are taken on the middle lines at the face of the sheet. The third graph is taken across the center of the sheet face. The mesh used for the simulation results depicted in Figure 4, Figure 5 and Figure 6 consists of 16604 tetrahedra. The duration of the simulation was 22.1 min on an Intel Xeon CPU E5-2630 v2 machine (2 processors) running at 2.6 GHz and with 39.1 GB of installed memory (RAM). Figure 4, Figure 5 and Figure 6 show very good uniformity of the magnetic field distribution and validate the model. The quality of the results was also validated by using denser and coarser meshes and comparing the simulation results for global variables. Thus, in the iterative procedure, the actual mesh consisted of 1222 tetrahedra. It should be noted that the air gap between the yoke and the electrical sheet steel has not been modeled, although the contact surfaces of the yoke and the tested strip are not perfectly smooth in reality. According to the standard, the maximum allowed air gap thickness is 5 μm. The attempts to introduce the air gap in the model failed, due to its very small thickness, compared to other dimensions. As well, and merely for the same reason, the air gap between the tested strip and the measuring winding has not been modeled. If the steel strip width is significantly smaller than the winding width, it must be modeled, which is easily achieved.

### 4.2. Simplorer Model with Transient Co-Simulation

**Figure 7:**
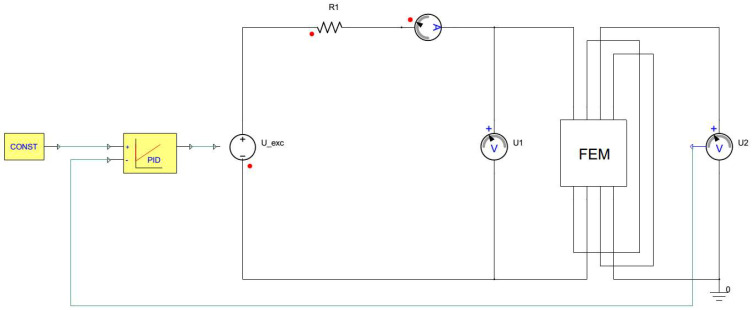
A Simplorer model with transient co-simulation with PI regulation for the excitation voltage waveform.

According to the aforementioned IEC standard, the shape of the magnetic induction waveform, i.e., the induced secondary voltage, needs to be sinusoidal [91]. That condition is fulfilled by the implementation of the standard PI regulator. The associated block diagram can be seen in Figure 3. The setpoint for the reference voltage of the regulator is sinusoidal with an amplitude corresponding to the required magnetic induction. The amplitude of the excitation voltage ${\hat{U}}_{exc}$ is calculated by the following Equation (18):

(18) $${\hat{U}}_{exc}=2\pi {fN}_{2}A_{Fe}\hat{B}$$

where f is the frequency, $A_{Fe}$ is the cross-section of the sample and $\hat{B}$ is the amplitude of the magnetic induction. The feedback for the PI regulator is the secondary voltage and the output of the regulator is the excitation voltage of the primary coil, where only the proportional term was used. The induced secondary voltage is proportional to the induction derivative, so the change of the induction derivative with respect to time is controlled. Figure 7 shows a co-simulation from Maxwell in the form of a black box with visible external pins; four input and four output terminals for the primary and secondary coils of the SST and for the primary and secondary coils of the air flux compensation coils.

## 5. Proposed Iterative Procedure for Error Correction

### 5.1. Steepest Descent Method and Gradient Estimation

In [22,23,24] an iterative procedure is applied for the correction of BH and power loss curves, using FEM and measurements on real electrical machines. The power loss function is corrected a posteriori, after correction of the BH curve. In both cases, the power loss curve and BH curve are parametrized, using three parameters for the BH curve and five parameters for the loss function, while the optimization is performed using the Levenberg–Marquardt method. Since we apply commercial Ansys Maxwell software, which performs the parametrization based on experimental data, defined in (B,H) and (P,B) discrete pairs, our task is somewhat different: instead of correcting the parameters, we correct the tabulated BH curve and loss pairs, and thus indirectly optimizing the parameters internally calculated by Ansys Maxwell. The task of correcting the BH curve and power loss curve is an unconstrained optimization problem of finding the minimum of the objective function *f*(***W***), where ***W*** denotes a vector of *N* parameters. The problem of functional minimization or maximization can be iteratively solved using several methods of nonlinear regression, including the Gauss–Newton method, steepest descent method, and the Levenberg–Marquardt method, where the Levenberg–Marquardt method is an interpolation technique between the Gauss-Newton and the steepest descent methods.

Instead of Levenberg–Marquardt method, which was applied in [22,23,24], in the sequel we will propose a different iterative procedure, based on the steepest decent method [92,93], alongside with the estimation of gradient based on quadratic cost function, applied in iteratively correction BH-curves and power loss curves. A comparison of Gauss-Newton, Levenberg–Marquardt and steepest descent methods, using a generalized mathematical framework, is given at the end of this subsection.

Starting from a given cost function

(19) $$f=f\left(w_{1},w_{2},\ldots ,w_{N}\right)$$

its extremum

(20) $$W^{*}=({w\prime }_{1},{w\prime }_{2},\ldots ,{w\prime }_{N})$$

is found iteratively in the steepest descent method evaluating partial derivatives at each step. Starting from the initial point $W^{\left(0\right)}$, a second point

(21) $$W^{\left(1\right)}={(w}_{1}^{\left(1\right)},w_{2}^{\left(1\right)},\ldots ,w_{N}^{\left(1\right)})$$

is found taking partial derivatives as

(22) $$W^{\left(1\right)}=W^{\left(0\right)}-\alpha \frac{𝜕f\left(W^{\left(0\right)}\right)}{𝜕w_{i}},i=1,2,\ldots ,N$$

where step size *α* is a small positive constant. In more compact matrix notation, we write Equation (22) as

(23) $$W^{\left(k\right)}=W^{\left(k-1\right)}-\alpha \nabla f(W^{\left(k-1\right)})$$

or

(24) $$W^{\left(k\right)}=W^{\left(k-1\right)}-\alpha g_{k}$$

where $g_{k}$ denotes a vector of partial derivatives at *k*-th iteration. We proceed with choosing the quadratic cost function

(25) $$f\left(W\right)=\sum_{i=1}^{N}{\left[d_{i}-y_{i}(W)\right]}^{2}$$

here $d_{i}$ denotes the desired output and $y_{i}$ denotes output of the model at *N* discrete points. This can be written in the matrix form as

(26) $$f\left(W\right)=\frac{1}{2}y^{T}Qy-y^{T}b$$

where ***y*** denotes a vector of model outputs and *T* denotes matrix transpose. Model outputs are vectors of power losses and peak currents, respectively. If analytical expressions for the derivatives are unavailable, the alternative is the application of their finite-difference approximations. Derivative $\frac{𝜕f\left(W^{k}\right)}{𝜕w_{i}}$ can be numerically approximated [94] by

(27) $$\frac{𝜕f\left(W^{\left(k\right)}\right)}{𝜕w_{i}}\approx \frac{f\left(w_{1},w_{2},\ldots ,w_{i}+h,w_{N}\right)-f(w_{1},w_{2},\ldots ,w_{N})}{h}$$

using some small h>0, which is a forward-difference approximation, or alternatively using central difference approximation [93], which is more exact but needs additional computations. The central difference approximation is defined by

(28) $$\frac{𝜕f\left(W^{\left(k\right)}\right)}{𝜕w_{i}}\approx \frac{f\left(w_{1},w_{2},\ldots ,w_{i}+h,w_{N}\right)-f(w_{1},w_{2},\ldots ,w_{i}-h,w_{N})}{2h}$$

while the backward difference is defined by

(29) $$\frac{𝜕f\left(W^{\left(k\right)}\right)}{𝜕w_{i}}\approx \frac{f\left(w_{1},w_{2},\ldots ,w_{i},w_{N}\right)-f(w_{1},w_{2},\ldots ,w_{i}-h,w_{N})}{h}$$

The central-difference approximation is more exact than forward differences but needs *N* additional function calculations. Both for forward differences and central differences, the truncation error can be limited to some extent by reducing the interval *h*. On the other hand, reducing the interval *h* increases the cancellation error, since quantities of similar magnitudes are subtracted [93]. Central differences are also more robust than forward differences, which may introduce numerical instability. The entries of the gradient vector of the chosen quadratic cost function are

(30) $$\frac{𝜕f\left(W^{\left(k\right)}\right)}{𝜕w_{i}}=2\left[d_{i}-y_{i}\right]\frac{𝜕y_{i}}{𝜕w_{i}}$$

Equation (26) can be applied to estimate the convergence of the iterative process and bounds of the step size *α*. For the BH curve, we evaluate *N u*-*i* pairs, where *u* and *i* are global variables, e.g., induced voltage at the secondary and the primary current. Voltages are adjusted in FEM to match measured values, and then corresponding currents and total losses are calculated. Total losses are calculated in FEM using Ansys internal routine, which apply Bertotti model, anhysteretic BH curve supplied with the model and non-homogenous distribution of magnetic flux density as result of numerical calculations. Note that the calculated losses depend both on the chosen BH curve and the parameters of the Bertotti model. Therefore, we may analyze the BH curve optimization independently from the correction of the power loss curve, while the correction of the power loss curve depends on the corrected BH curve. For power loss correction, a good estimate of the FEM calculated power loss is the input power loss curve, which leads to y≈W. The same holds for the BH curve correction, where a good estimate of the calculated BH curve is the input BH curve. In this way, Equations (30) and (26) become approximated as

(31) $$\frac{𝜕f\left(W^{\left(k\right)}\right)}{𝜕w_{i}}\approx 2\left[d_{i}-y_{i}\right]$$

(32) $$f\left(W\right)\approx \frac{1}{2}W^{T}QW-W^{T}b$$

where ***Q*** = 2·I, and ***I*** denotes an N×N identity matrix. Regarding the convergence, for a cost function *f*(***W***) convex and differentiable, satisfying the first-order β-Lipschitz condition, the cost function at the *k*-th iteration is related to the minimizer $W^{*}$ as

(33) $$f\left(W^{\left(k\right)}\right)-f\left(W^{*}\right)\leq \frac{\beta }{2(k+1)}\left|W^{\left(0\right)}-W^{\left(k\right)}\right|$$

where $W^{\left(0\right)}$ denotes initial point and β=1/α where α denotes step size [92]. For strongly convex cost functions, the convergence speed can be increased from arithmetic to geometric or linear convergence. For the quadratic cost function, the optimal value of step size $\alpha_{k}$ can be derived at *k*-th step explicitly as [92]

(34) $$\alpha_{k}=\frac{g_{k}^{T}g_{k}}{g_{k}^{T}Qg_{k}}$$

Assuming approximation of ***Q*** according to (31) and (32), we arrive at the step size equal to α=1/2. Introducing an auxiliary function

(35) $$E\left(W\right)=\frac{1}{2}\left(W-W^{*}\right)Q(W-W^{*})$$

The bound for the rate of convergence is, following the Kantorovich inequality [92,95]

(36) $$\frac{E\left(W^{\left(k\right)}\right)-E\left(W^{\left(k+1\right)}\right)}{E\left(W^{\left(k\right)}\right)}=\frac{{\left(g_{k}^{T}g_{k}\right)}^{2}}{\left(g_{k}^{T}Qg_{k})(g_{k}^{T}Q^{-1}g_{k}\right)}\geq \frac{4aA}{{\left(A+a\right)}^{2}}$$

where *A* denotes the largest and *a* the smallest eigenvalue of ***Q***.

**Figure 8:**
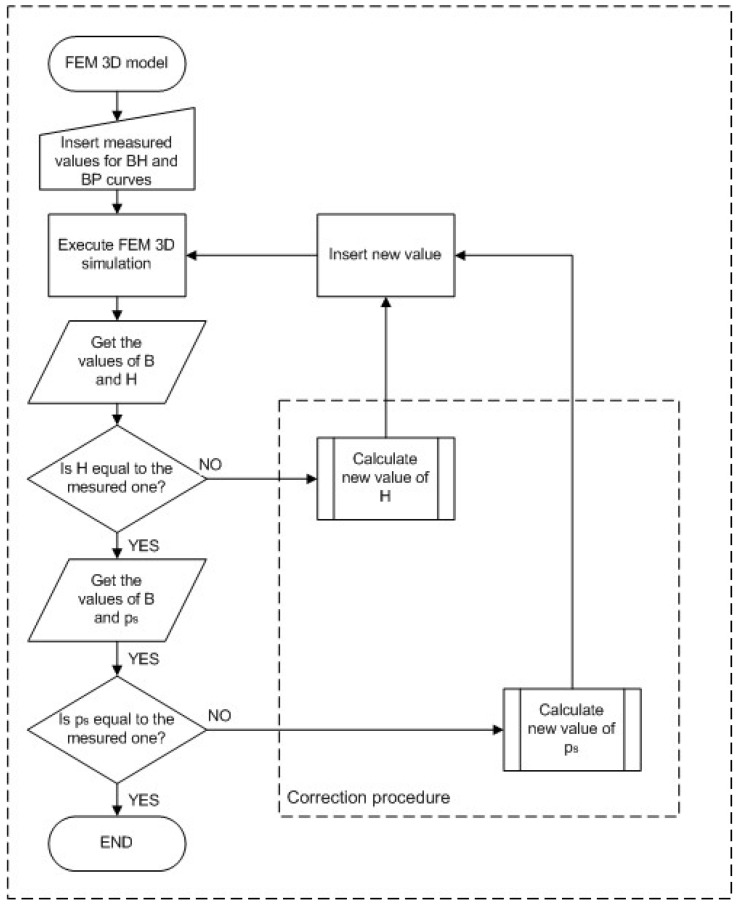
A general flowchart of an iterative procedure for error correction in BH and PB curves.

A general flowchart of the iterative procedure that was used to calculate the final values of magnetic field strength and core losses of an electrical steel sheet is shown in Figure 8. Each iteration results with modified BH and PB curves, which are then implemented back into FEM. These iterative procedures were applied to the point where the simulation errors in the FEM are significantly reduced, and the simulation results are more consistent with the measured values.

Steepest descent methods, using first-order derivatives, quickly converge to the solution from remote points, but their convergence slows near the solution. Gauss-Newton and steepest descent algorithms can be described by general formula

(37) $$W^{\left(k\right)}=W^{\left(k-1\right)}-\alpha {M^{\left(k\right)}g}_{k}$$

the $M^{\left(k\right)}$ denotes an N×N matrix. For the steepest descent method holds $M^{\left(k-1\right)}=I$, where ***I*** denotes the identity matrix. For the Gauss–Newton method, $M^{\left(k\right)}={\left[G^{\left(k\right)}\right]}^{-1}$, $G^{\left(k\right)}$ is the second derivative (Hessian) N×N matrix of objective function evaluated at $W^{\left(k-1\right)}$. The Gauss–Newton method approximates locally a function that is being minimized by a quadratic function, using a truncated Taylor series. This method is well defined near the solution and has very desirable properties if started sufficiently close to the solution [92]. However, it must be modified to be applied at remote points, where positive definiteness of the matrix of second derivatives (and convergence of the process) is not assured. A common solution is taking $M^{\left(k\right)}={\left[{\varepsilon_{k}I+G}^{\left(k\right)}\right]}^{-1}$, where $\varepsilon_{k}$ denotes a non-negative constant. For $\varepsilon_{k}=0$, the method degenerates to Gauss–Newton method, while for very large values of $\varepsilon_{k}$, this approach leads to the steepest descent methods, while $M^{\left(k\right)}$ can always be made positive-definite by proper solution of $\varepsilon_{k}$. For a given $\varepsilon_{k}$, in the Levenberg–Marquardt method positive definiteness of ${\varepsilon_{k}I+G}^{\left(k\right)}$ is checked using Cholesky decomposition. If Cholesky decomposition collapses, $\varepsilon_{k}$ is increased. In such a way, the Levenberg–Marquardt method combines the Gauss–Newton method and steepest descent methods, acting like steepest descent for remote points and Gauss-Newton for points close to the optimal value. The steepest descent method converges rapidly at the first few iterations, but then ends in an oscillatory behavior (zigzagging) [93]. The Newton-type methods require the second-order derivatives, which are computationally more demanding, and better converge near the solution. For completeness, the adjoint-state method for the gradient computation should be mentioned [96]. This method can be used for partial differential equations-constrained optimization problems, and the adjoint state space simplifies the interpretation of the constraints and makes the calculation of the gradient very fast.

### 5.2. Error Correction Method for BH Curve

Prior to the iterative correction method, the induced secondary voltages ${U_{2x}}^{0}$ and magnetizing currents ${I_{1x}}^{0}$ need to be calculated from the experimental (${B_{x}}^{0}$,${H_{x}}^{0}$) discrete pairs and the parameter values of the SST apparatus as follows in (38) and (39):

(38) $${U_{2x}}^{0}=\frac{N_{2}A\omega }{\sqrt{2}}{B_{x}}^{0}$$

(39) $${I_{1x}}^{0}=\frac{l_{ef}}{N_{1}}{H_{x}}^{0}$$

here, value zero in the upper indexes of ${U_{2x}}^{0}$, ${I_{1x}}^{0}$, ${B_{x}}^{0}$ and ${H_{x}}^{0}$ denote experimental data, x denotes specific (${B_{x}}^{0}$,${H_{x}}^{0}$) discrete pair, $N_{1}$ and $N_{2}$ are number of turns of the primary and secondary coil, respectively, A is inner core area of the secondary coil, $l_{ef}$ is effective magnetic path length on the primary side and ω is angular frequency.

**Figure 9:**
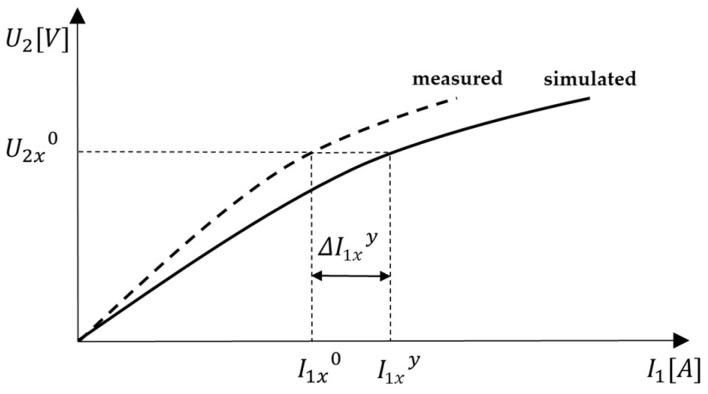
Graphical representation of error correction for magnetization current.

Then, in every iteration, the correction method is applied on each discrete pair of the BH curve. The correction values of the magnetizing current ${{\Delta I}_{11}}^{y}$, ${{\Delta I}_{12}}^{y}$...$\;{{\Delta I}_{1x}}^{y}$ are calculated as follows in (40), where ${I_{11}}^{y}$, ${I_{12}}^{y}$... ${I_{1x}}^{y}$ are the peak values of the magnetizing current in $y^{th}$ iteration (y=1, 2, 3…) and these are the output results of FEM simulation. The graphical representation of the mentioned procedure can be seen in Figure 9.

(40) $$\begin{array}{c} {{\Delta I}_{11}}^{y}={I_{11}}^{y}-{I_{11}}^{0}\\ {{\Delta I}_{12}}^{y}={I_{12}}^{y}-{I_{12}}^{0}\\ \vdots \\ {{\Delta I}_{1x}}^{y}={I_{1x}}^{y}-{I_{1x}}^{0} \end{array}$$

In the following step, the correction values for the magnetic field strength ${{\Delta H}_{x}}^{y}$ are calculated as it is shown in (41), while the values for each (${B_{x}}^{y+1}$,${H_{x}^{\prime }}^{y+1}$) discrete pair that are used in the next $({y+1)}^{th}$ iteration as an inputs for FEM simulation, are given in (42):

(41) $${{\Delta H}_{x}}^{y}=\frac{N_{1}}{l_{ef}}{{\Delta I}_{1x}}^{y}$$

(42) $${H_{x}^{\prime }}^{y+1}={H_{x}^{\prime }}^{y}-{{\Delta H}_{x}}^{y}$$

where the ${H_{x}^{\prime }}^{y+1}$ and ${H_{x}^{\prime }}^{y}$ are corrected values of the magnetic field strength that are written in input BH table which is used for FEM simulations in ${\left(y+1\right)}^{th}$ and $y^{th}$ iterations. Equations (40) and (41) follow from the optimal step size α=1/2 and Equations (26) and (31), i.e., $w_{i}^{\left(k\right)}\approx w_{i}^{\left(k-1\right)}+\alpha \cdot 2\left[d_{i}-y_{i}\right]=w_{i}^{\left(k-1\right)}-\left[y_{i}-d_{i}\right]$.

The iterative process is repeated until the simulation results of the BH curve fit within the predefined error. The comparison of the results was based on the root-mean-square error (*RMSE*) criterion (43):

(43) $$RMSE=\sqrt{\frac{\sum_{x=1}^{N}{\left({H_{x}}^{y}-{H_{x}}^{0}\right)}^{2}}{N}}$$

here, the ${H_{x}}^{y}$ are simulation output results of the magnetic field strength in $y^{th}$ iterations and N is total number of (BH) discrete pairs.

### 5.3. Error Correction Method for PB Curve

The minimization of errors in the BH curve has a significant impact on the error reduction in PB curves in the whole range of magnetic induction. Further steps that bring simulated results of total magnetic losses closer to the measured include methods like the one that has been described above. At a certain magnetic induction, the value of the magnetic loss is directly calculated using the iterative procedure.

**Figure 10:**
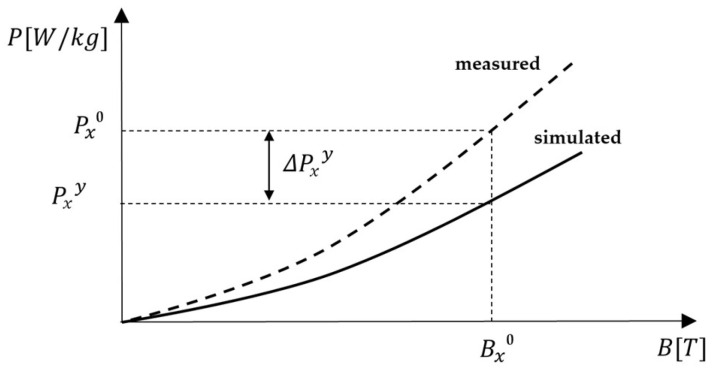
Graphical representation of magnetic loss error correction.

The values ${{\Delta P}_{x}}^{y}$ that represents the correction of the magnetic losses in each point of the PB curve is calculated as follows in (43), where ${P_{1}}^{y}$, ${P_{2}}^{y}$... ${P_{x}}^{y}$ are simulation results of the magnetic losses in $y^{th}$ iteration (y=1, 2, 3…), while ${P_{1}}^{0}$, ${P_{2}}^{0}$... ${P_{x}}^{0}$ are experimental values of (${P_{x}}^{0},{B_{x}}^{0}$) discrete pairs. The procedure for one iteration at a point x could be seen in Figure 10 and Equation (44):

(44) $$\begin{array}{c} {{\Delta P}_{1}}^{y}={P_{1}}^{y}-{P_{1}}^{0}\\ {{\Delta P}_{2}}^{y}={P_{2}}^{y}-{P_{2}}^{0}\\ \vdots \\ {{\Delta P}_{x}}^{y}={P_{x}}^{y}-{P_{x}}^{0} \end{array}$$

The values for each (${P_{x}^{\prime }}^{y+1},{B_{x}}^{y+1}$) discrete pair that are used in the next $({y+1)}^{th}$ iteration as an input for FEM simulation, are given in (45).

(45) $${P_{x}^{\prime }}^{y+1}={P_{x}^{\prime }}^{y}-{{\Delta P}_{x}}^{y}$$

here, ${P_{x}^{\prime }}^{y+1}$ and ${P_{x}^{\prime }}^{y}$ are corrected values of the magnetic losses that are written in the input PB table which is used for FEM simulations in ${\left(y+1\right)}^{th}$ and $y^{th}$ iterations.

The error reduction was evaluated with the same criterion as in the case of the BH curve procedure, the *RMSE* (46)

(46) $$RMSE=\sqrt{\frac{\sum_{x=1}^{N}{\left({P_{x}}^{y}-{P_{x}}^{0}\right)}^{2}}{N}}$$

here, ${P_{x}}^{y}$ are simulation output results of the magnetic losses in $y^{th}$ iterations and N is total number of (BH) discrete pairs.

## 6. Results

### 6.1. Measurements

The measurements were performed using a customized single-sheet tester with measuring unit MPG 200 D (Brockhaus, Lüdenscheid-Germany). The customized sheet tester is designed to accommodate sheet samples with dimensions 210 mm × 210 mm. The samples were sheets (210 mm × 210 mm) of the grain-oriented electrical steel sheet, which is commonly used in the industry of highly efficient electrical machines.

Maintaining the same quadratic ratio of the samples, as in standard 500 mm × 500 mm SST with scale ratio 2.1/5, and taking into account the small width of the sample, it is reasonable to consider measurements as representative for the models of upscaled standard 500 cm × 500 cm SST. This assumption was confirmed using a down-scaled FEM of tester and using the same thickness of the sample (0.2161 mm). Table 2 gives the differences between scaled FEM (210 mm × 210 mm) in comparison to 500 mm × 500 mm SST. Note that this relative difference can be applied only to this particular material and thickness of the sample and does not represent a general rule. For different materials, ratios and dimensions of samples, those differences may vary [97]. On the other side, SST measurements performed with different equipment of the same size show high reproducibility (standard deviation <2%  for non-oriened steel and <1%  for grain-oriented steel). According to the manufacturer’s data sheet, adjustment of the nominal value of polarization (selectable from 1 mT to 2 T) is better than 0.1%, while the repeatability is better than 0.2%. To estimate the uncertainty of measured magnetic flux density *B*, we apply a conservative approach assuming limit errors $a_{a\%}=0.1\%$ and $a_{r\%}=0.2\%$ and rectangular distribution of errors. We arrive at associated uncertainties $u_{a}=a_{a\%}\frac{B}{100\sqrt{3}}$ and $u_{r}=a_{r\%}\frac{B}{100\sqrt{3}}$. Using the same conservative approach, we combine $u_{a}$ and $u_{r}$ to obtain the estimation of expanded (coverage factor *k* = 2, confidence level 95%) relative uncertainties of measured magnetic field *H*:

(47) $$U_{H\%}=\frac{2B}{\sqrt{3}\mu_{diff}H}\sqrt{a_{a\%}^{2}+a_{r\%}^{2}}$$

Power loss is dependent on magnetic flux density and frequency. The adjustment of the nominal frequency is according to the manufacturer’s data sheet better than $a_{f\%}=$ 0.2%. We arrive again, assuming the rectangular distribution of errors

(48) $$u_{P\%}=\frac{B\;dP/dB}{\sqrt{3}}\sqrt{a_{a\%}^{2}+a_{r\%}^{2}}$$

(49) $$u_{f\%}=\frac{f\;dP/df}{\sqrt{3}}a_{f\%}$$

which gives expanded combined relative uncertainty (*k* = 2):

(50) $$U_{f\%}=\frac{2}{P}\sqrt{u_{P\%}^{2}+u_{f\%}^{2}}$$

The sensitivity coefficient for the frequency was calculated using measurements of power loss at frequencies 50 Hz and 60 Hz, as dP/df≈∆P/∆f. Differential permeability $\mu_{diff}=dB/dH$ and derivative dP/dB were calculated numerically, using central difference approximation from measured *B*, *H*, and *P*, except for the last term, which was estimated using backward differences. Estimated uncertainties $U_{H\%}$ and $U_{P\%}$ are presented alongside the results in Table 3 and Table 4. The measurement results were processed and stored using professional SST software v4.0, MPG-Expert 3.x (Brockhaus), which is a control interface for PC driven measuring systems for the determination of magnetic properties of soft magnetic materials.

### 6.2. Corrected Curves

**Figure 11:**
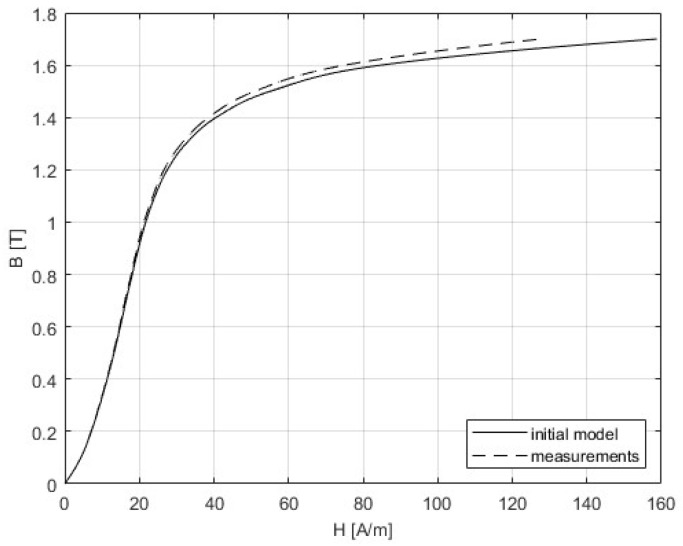
Comparison of the measurements and the initial model for the BH curve. The curve representing the initial model is obtained using FEM and global variables.

**Figure 12:**
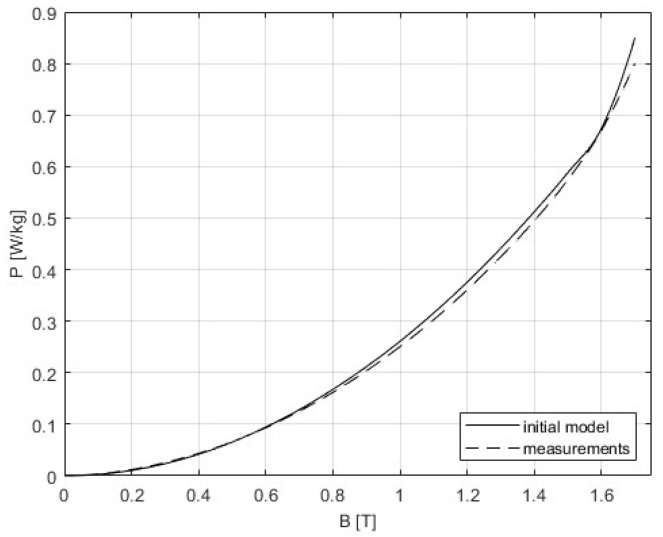
Comparison of the measurements and the initial model for the PB curve. The curve representing the initial model is obtained using FEM.

**Figure 13:**
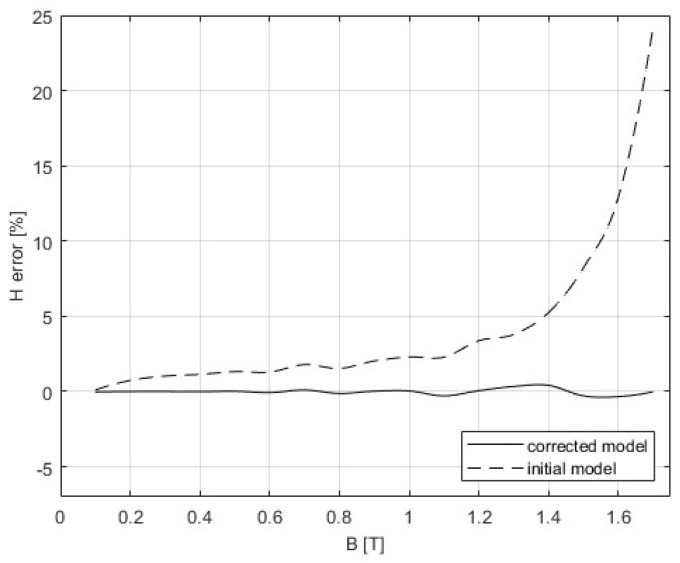
Comparison of the relative errors for the initial and corrected model for the BH curve.

**Figure 14:**
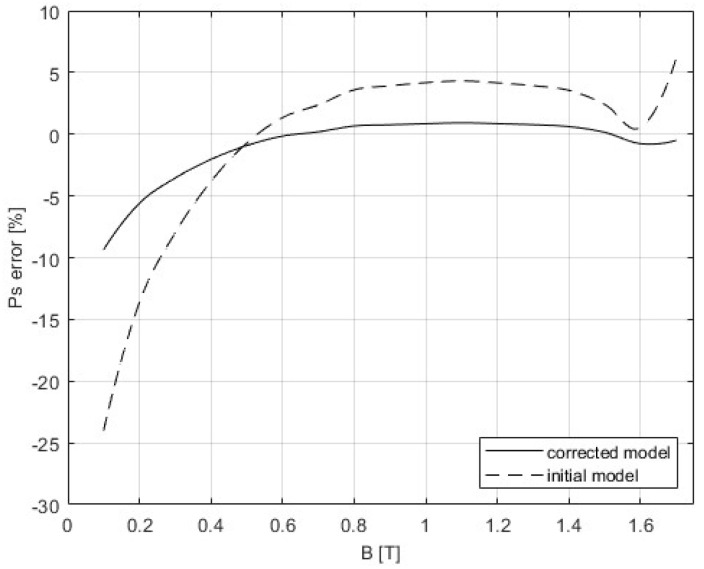
Comparison of the relative errors for the initial and corrected model for the PB curve.

**Figure 15:**
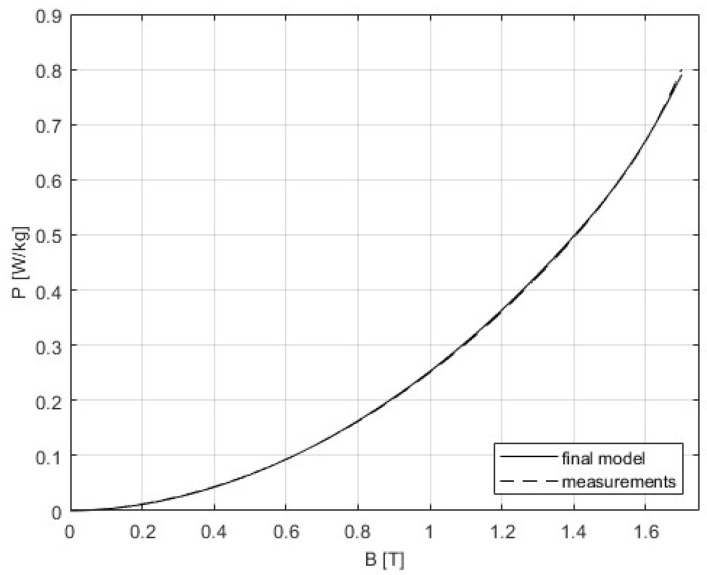
Comparison of the measurements and corrected model for the PB curve. The curve representing the corrected model is obtained from FEM.

**Figure 16:**
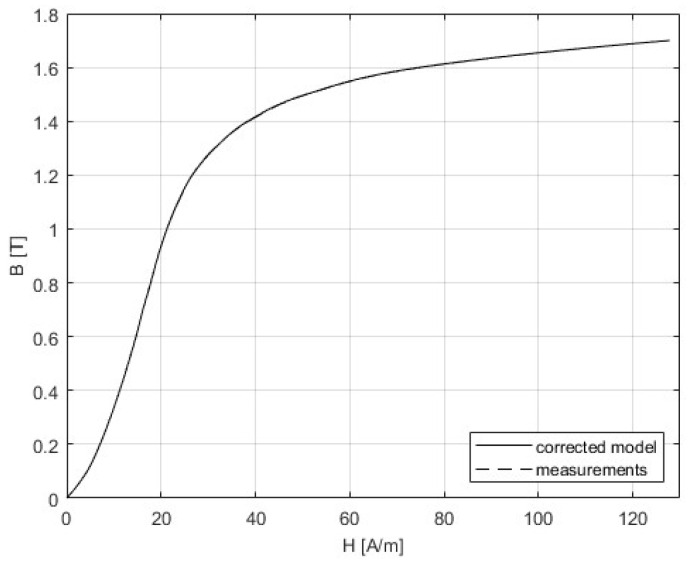
Comparison of the measurements and corrected model for the BH curve. The curve representing the corrected model is obtained using FEM and global variables.

The 3D SST model is built as it is described in Section 4, and the correction procedure from Section 5 was applied. The following figures present the results that were obtained from the initial simulation and after the BH and PB curves were corrected. The difference between measurements and modeling could be seen in Figure 11 and Figure 12, while Table 5 gives a comparison of the results of the convergence process based on the RMSE criterion. Graphs showing the relative errors for the initial and corrected models for BH and PB are presented in Figure 13 and Figure 14, respectively, while Table 3 and Table 4 detail the relative errors (expressed as percentages) in relation to the measured data. Comparison of the measurements and corrected models is presented in Figure 15 and Figure 16. The measurements for the BH and PB curves were conducted within a magnetic flux density range of 0.1 to 1.7 T, incremented by 0.1 T, resulting in a total of 17 data points. These measurements were performed along the rolling direction at a frequency of 50 Hz. Depending on the sample characteristics, the number of iterations needed in our numerical experiments with different materials varied between 1 and 4, which is expected for the steepest descent method. In this example, the minimum was reached in only one iteration. Because of the known oscillatory behavior of the steepest descent method near the solution, after reaching a minimum for the first time, the next iteration might worsen the residual error. For the easy comparison of the results, the measured BH curves are shown as defined by the standard IEC 60404-3, calculated from secondary voltage and primary current (global variables) and extracted from the manufacturer’s software. The curves related to the model are calculated in the same manner from FEM of the device. Note that both curves are always related to the currents and voltages as global variables, although magnetic flux densities and the strength of magnetic fields are marked as variables. To avoid possible misinterpretation, the corrected BH curve itself is deliberately omitted. The power losses are defined in SST measurement using global variables as [8]

(51) $$P=\frac{N_{1}}{{(N}_{2}Sl_{m}\delta )}\int_{T}^{⁠}u\left(t\right)i(t)dt$$

where $N_{1}$ is the primary turn number, $N_{2}$ is the secondary turn number, *S* is the cross-section of the specimen, $l_{m}$ is the magnetic path length and δ is the density of the specimen. Furthermore, $u\left(t\right)$ and i(t) denote induced voltage and primary current. Due to the single-valued, anhysteretic model of the BH curve, power losses in the FEM are calculated internally in Ansys Maxwell using local values of the magnetic field densities and Bertotti model of power losses.

In [24], relative errors in recovered characteristics were defined as ratios between the area under the recovered and the original characteristics. Therefore, we further evaluate our results (based on global variables) in comparison to those presented in [24] using errors defined by the following formulae:

(52) $$E_{BH}={\left(\frac{\int_{0}^{H_{max}}B_{recovered}dH}{\int_{0}^{H_{max}}B_{original}dH}-1\right)}^{2}\times 100\%$$

(53) $$E_{PB}={\left(\frac{\int_{0}^{B_{max}}P_{recovered}dB}{\int_{0}^{B_{max}}P_{original}dB}-1\right)}^{2}\times 100\%$$

$E_{BH}\;$ is defined by Equation (51) in [98], and quoted in [24], while $E_{PB}$ is defined in [24] only in text, without an explicit formula. In [23], $B_{original}$ and $P_{original}$ were measured using the stator core of the same commercial induction motor, which was previously used for the measurements of the global variables (i.e., the excitation current and the voltage over the main excitation windings). For those ‘original’ measurements, the following modifications were made: the rotor was removed, and extra excitation and measurement windings were wound around the stator. In such a way, a ring-type core was constructed from the stator core, approximating the IEEE standard for testing magnetic ring cores. Based on the measurements and simulations of global variables and neglecting the stator-end windings in 2D FEM simulations, the recovery error $E_{BH}\;$ was 1.45% [23]. Using a different objective function, using measured local peak magnetic fluxes (less affected by stator end-winding and measured by inserted search coils), a lower recovery error was achieved (0.11%). Since our measurements were performed on a single-sheet sample, direct comparison with the ring-core measurements was not possible. Nevertheless, we informatively supply our recovery errors treating SST measurements as $B_{original}$ and simulated global variables as $B_{recovered}$, derived using Equations (38) and (39). The recovery error $E_{BH}\;$ was $2.032\cdot 10^{-5\;}\;\%$ (after correction), while initial error $E_{BH}\;$ between FEM and measurements was 0.026%. Since our curves (initial and corrected) are in discrete pairs, a smoothing spline fit was performed in MATLAB R2022b using the command fit, and the fit object was integrated using the MATLAB command integrate. All our errors associated with BH curves were calculated using $H_{max}=127\;$ A/m. Furthermore, it should be noted that in [24], the recovery errors were not calculated directly against the measurements. Instead, the original BH curve measurements (using a “ring-core” setup made from the stator) were fit to its three-parameter approximation:

(54) $$\frac{H}{H_{0}}={\left(\frac{B_{⁠}}{B_{0}}\right)}^{⁠}+{\left(\frac{B_{⁠}}{B_{0}}\right)}^{\nu }$$

and the fitted model was used as the original curve. As well, the same three-parameter approximation was used as the model of the BH curve in the correction procedure, where model parameters ($H_{0},\;B_{0},\;\nu )$ were calculated using an inverse approach. Using our SST measurements of BH pairs, the three-parameter approximation (54) of our BH data introduces a modeling error equal to $2.8\cdot 10^{-3\;}\%$, which is significantly greater than our recovery error $E_{BH}\;$(which was $2.032\cdot 10^{-5\;}\%$). This modeling error was calculated using (51), while the parameters ($H_{0},\;B_{0},\;\nu )$ were calculated using *Curve Fitter* in MATLAB. Remark: since three-parameter approximation gives *H* as a function of *B*, its inverse was obtained as a MATLAB fit object, using smoothing spline approximation. In a similar manner, we have calculated $E_{PB}$ errors (using $B_{max}=1.7\;T)$, with the following results: the recovery error was 0.0035%, while the initial error was 0.0307%. Note that our procedure, as well as the approach presented in [24], utilizes the Bertotti loss model, which introduces similar modeling errors in both approaches. The recovery error $E_{PB}$ in [24] was 0.49% at 50 Hz, with the same disclaimer that the comparison was made against “ring-core” measurements.

## 7. Discussion and Conclusions

The inhomogeneity of the magnetic field is one of the factors which has a great influence and causes the spatial deviation of the magnetic field, especially at high magnetic inductions, resulting in the discrepancies between FEM simulations and simplified analytical magnetic equivalent circuit approach. Due to the saturation effect at higher magnetic flux densities, the differences increase. Since the standardized measurement of the BH curve and magnetic losses rely on the simplified analytical approach, the results of the initial standard FEM 3D model deviate from measurements by up to 24% for the selected type of steel sheets if such kind of measured material characteristics were applied in the model. To enhance the measurement results, a correction procedure was applied through a series of iterative corrections of material characteristics, and the difference between the model and measurements was significantly reduced.

The proposed iterative correction procedure is based on the steepest descent algorithm, while the stopping criteria were based on the difference between simulated and measured global variables (power loss, induced voltage, and primary current). Since the proposed gradient estimation leads to its simple calculation, it is possible to perform easy updating of a relatively large number of parameters (17 or 20) used in optimization at each step. This, in turn, allows the indirect correction of the BH curve and loss curve using the commercial Ansys Maxwell software, which internally performs the parametrization based on user-defined data, supplied in (B,H) and (B,P) discrete pairs. After correction, root mean squared errors were decreased from 1.85 A/m to 42.9 × 10^−3^ A/m for the BH curve, and from 44.5 × 10^−4^ W/kg to 7.3 × 10^−4^ W/kg for the power loss curve.

For the initial model, the deviation of the magnetic field strength from experimental data increases with magnetic flux density. The errors grow approximately linearly up to 1 T, after which they increase exponentially. Between 0.1 T and 1 T, the relative errors range from 0.1% to 2.3%, reaching a maximum of approximately 24.2% at 1.7 T. After applying the correction method, the errors are significantly reduced across the entire range of magnetic flux density, with values remaining within ±0.34%.

When total magnetic losses are considered, the highest relative errors occur at low magnetic flux densities, specifically below 0.4 T, with a maximum deviation of −24% at 0.1 T. Between 0.4 T and 1.7 T, the relative errors remain within ±5%. When both corrected BH and PB curves are incorporated into the model, the results improve significantly. Although the highest error still occurs at low magnetic flux densities, it is reduced to approximately −9.4% at 0.1 T and decreases across the entire range up to 1.7 T. For magnetic flux densities above 0.5 T, the errors are within ±1%. The larger residual errors at low magnetic flux densities might suggest the existence of unmodeled excess loss due to the pinning effect and domain wall bowing [99,100]. The additional experiments should give a clearer physical explanation of this effect. This specific example shows that the correction works properly. For different geometric details, a new FEM has to be built to accompany new measurements results and make the corrections. However, if geometric details are the same, the same model can be used with new measurements; only the parameters defining the BH curve and PB curve must be replaced with a new set of data.

## Figures and Tables

**Table 1:** Dimensions of the SST and related parameters.

Parameter	Symbol	Value
Sample length [mm]	l	700
Sample width [mm]	w	500
Sample thickness [mm]	d	0.2161
$\mathrm{Sample}\;\mathrm{density}\;[\frac{kg}{m^{3}}$]	δ	7650
Yoke’s length [mm]	$l_{y}$	500
Yoke’s width [mm]	$w_{y}$	500
Yoke’s height [mm]	$h_{y}$	120
Yoke’s thickness [mm]	$d_{y}$	25
Primary coil turns	$N_{1}$	400
Secondary coil turns	$N_{2}$	400

**Table 2:** Relative differences—scaled FEM of SST device in comparison to 500 mm × 500 mm FEM.

B [T]	0.1	0.5	1.0	1.5	1.7
H difference [%]	0.639	1.195	1.642	0.798	0.365

**Table 3:** Relative errors for the initial and corrected model for the BH curve alongside with estimated expanded relative uncertainties *U* of measurements (*k* = 2).

B [T]	0.1	0.2	0.3	0.4	0.5	0.6	0.7	0.8	0.9	1.0	1.1	1.2	1.3	1.4	1.5	1.6	1.7
H init [%]	0.105	0.738	1.028	1.138	1.322	1.295	1.79	1.536	2.0298	2.295	2.286	3.373	3.796	5.247	8.218	12.886	24.244
H corr [%]	−0.023	0.001	0.004	−0.008	0.02	−0.071	0.103	−0.127	0.024	0.034	−0.289	0.055	0.342	0.411	−0.29	−0.34	−0.012
U est [%]	0.211	0.181	0.176	0.173	0.171	0.165	0.175	0.187	0.205	0.261	0.322	0.446	0.640	0.919	1.389	2.116	1.812

**Table 4:** Relative errors for the initial and corrected model for PB curve alongside with estimated expanded relative uncertainties *U* of measurements (*k* = 2).

B [T]	0.1	0.2	0.3	0.4	0.5	0.6	0.7	0.8	0.9	1.0	1.1	1.2	1.3	1.4	1.5	1.6	1.7
P init [%]	−24.01	−13.64	−7.98	−3.82	−0.71	1.34	2.37	3.59	3.92	4.17	4.31	4.16	3.93	3.55	2.42	0.54	6.18
P corr [%]	−9.36	−5.6	−3.55	−2.041	−0.92	−0.15	0.19	0.66	0.76	0.85	0.92	0.86	0.77	0.62	0.16	−0.76	−0.5
U est [%]	0.514	0.564	0.582	0.592	0.604	0.611	0.617	0.626	0.631	0.634	0.640	0.643	0.645	0.656	0.690	0.779	0.791

**Table 5:** Comparison of the initial and corrected models based on the RMSE criterion.

	BH Curve	PB Curve
	RMSE [A/m]	RMSE [W/kg]
Initial model	1.84673	$44.5\;\cdot 10^{-4}$
Corrected model	42.88$\;\cdot 10^{-3}$	$7.27791\;\cdot 10^{-4}$

## Data Availability

The data presented in this study are available from the corresponding author upon request.
